# Editorial: Follicular Helper T Cells in Immunity and Autoimmunity

**DOI:** 10.3389/fimmu.2020.01042

**Published:** 2020-05-29

**Authors:** Maria Pia Cicalese, Shahram Salek-Ardakani, Georgia Fousteri

**Affiliations:** ^1^San Raffaele Telethon Institute for Gene Therapy (TIGET), San Raffaele Scientific Institute, Milan, Italy; ^2^Pediatric Immunohematology and Bone Marrow Transplantation Unit, IRCCS San Raffaele Scientific Institute, Milan, Italy; ^3^Department of Pathology, Immunology and Laboratory Medicine, University of Florida, Gainesville, FL, United States; ^4^Division of Immunology Transplantation and Infectious Diseases (DITID), Diabetes Research Institute (DRI), IRCCS San Raffaele Scientific Institute, Milan, Italy

**Keywords:** follicular helper T cells, follicular regulatory T cells, autoimmunity, primary immunodeficiency, immunity, cancer, infection, transplantation

In the last two decades, a new population of CD4^+^ helper T cells, named T follicular helper cells (Tfh), was shown to be specialized in “helping” the germinal center (GC) response and reported to play important roles in type I, II, and III immune responses. High expression of CXCR5 and low expression of CCR7 enable Tfh cells to enter and stay in GCs. In the light zone of GCs, Tfh cells provide crucial signals to antigen-specific B cells, promoting somatic hypermutation, class switch recombination (CSR), and affinity maturation through cellular interactions and cytokine secretion. In addition, Tfh cells also facilitate the differentiation of memory B cells and long-lived plasma cells. Tfh cells can be found in circulation and recent studies have identified multiple Tfh subsets based on the expression of chemokine receptors and activation molecules. The distribution of circulating Tfh subsets, their number and activation phenotype have been associated with the clinical outcome of numerous diseases spanning from autoimmunity to immunodeficiency and were shown to correlate with responses to infections and vaccines (1). Furthermore, a population of FOXP3^+^ regulatory T cells that expresses CXCR5 and controls GC responses, named follicular regulatory T cells (Tfr)s, was recently described and shown to suppress Tfh cell-mediated antibody responses. However, the factors that control their generation and the mechanisms of suppression are poorly understood (2).

Tfh and Tfr cells are currently being used as biomarkers and therapeutic targets in various clinical settings, including cancer and transplantation (3–13). A clear understanding of the mechanisms that control the development of Tfh and Tfr cells and the factors that contribute to GC responses is vital to the success of Tfh-based immune-monitoring and therapy development. This Special Research Topic aimed to address several questions with the final objective to understand better the biology of Tfh cells and how they could be targeted to harness undesired immune responses or boost immunity. Our Special Research Topic “Follicular Helper T Cells in Immunity and Autoimmunity” brought together several outstanding experts in the field. Here, we discuss the main messages from eight original research articles, one perspective and twenty state-of-the-art review articles these experts contributed divided into eight themes, as reported below.

## Molecular and Cellular Regulation of Tfh Cell Development and Differentiation

The development and differentiation of Tfh cells have been shown to be regulated by transcription factors such as the B-cell lymphoma 6 protein (Bcl-6), the signal transducer and activator of transcription 3 (STAT3), and the B lymphocyte-induced maturation protein-1 (Blimp-1). Also, cytokines, including IL-6 and IL-21, are essential for Tfh cell development. In the last step of Tfh cell commitment, B cells become the primary antigen-presenting cell and provide signals including ICOSL/ICOS, CD40/CD40L, and CD84/CD84-SAP that complete Tfh cell differentiation program (1). In their review article, Wu et al. summarize the recent advances in the molecular regulation of Tfh cell development and differentiation at the protein and epigenetic levels. Identification of the factors that instruct Tfh cell differentiation could increase the pipeline of potential targets for future clinical interventions.

On the other hand, Tfh cell dysregulation may result in aberrant antibody responses that frequently coincide with autoimmune disease or allergy development (4). The fate and identity of Tfh cells are tightly controlled by gene regulation at the transcriptional and post-transcriptional level. Baumjohann et al. provide a nice overview on the complex and dynamic regulatory network of post-transcriptional mechanisms that regulate Tfh cell differentiation, function, and plasticity through the actions of RNA-binding proteins (RBPs, belonging to the Roquin family) and small endogenously expressed regulatory RNAs called microRNAs (miRNAs). RBPs have been shown to dampen spontaneous activation and differentiation of naïve CD4^+^ T cells into Tfh cells, as evidenced by the propensity of Roquin-mutant CD4^+^ T cells to differentiate into Tfh cells providing inappropriate B cell help signals promoting the production of autoantibodies. Interestingly, Regnase-1, an endoribonuclease that regulates many molecules that are also targets of Roquin, was crucial for the prevention of autoantibody production (Baumjohann et al.).

Significant progress has been made in defining the potential of different dendritic cell (DC) subsets in supporting Tfh priming (Wu et al.). Krishnaswamy et al. propose that the location of different DC subsets within the lymph node (LN) and their access to antigen determines their potency in Tfh cell priming. Indeed, migratory cDC1s, resident cDC1s, and Langerhans cells (LCs) are found within the T cell zones. Migratory cDC2s are located in the T-B border region (including the interfollicular zone) and resident cDC2s reside in the lymphatic sinuses. The authors propose an interesting three-step model for Tfh cell differentiation involving diverse subpopulations of DCs: step 1: Antigen transport and naïve T cell activation (antigen presented by migratory cDCs, LN-resident cDC2s or transported by migratory DCs to resident DC subsets in the LNs); step 2: Pre-Tfh differentiation by migratory cDC2s at the T-B border; step 3: Tfh commitment by B cells (Krishnaswamy et al.).

A very elegant summary of the GC niche and the complex immunological synapses between Tfh and GC B cells are provided by Papa and Vinuesa. According to the authors, Tfh:B cell cognate interactions need to be critically fast and short at the level of the GC for affinity-selection, where in B cells compete for T cell help so that rapid modulation of the signaling threshold determines the outcome of the interaction. Moreover, promiscuous or bystander delivery of positive selection signals could potentially lead to the appearance of long-lived self-reactive B cell clones. Cytokines, cytotoxic granules, and more recently neurotransmitters including dopamine were shown to be part of Tfh-B cell interaction and the involvement of a much larger array of neurotransmitter-like molecules is expected to be discovered in the following years (Papa and Vinuesa).

## Tfh Cells in Autoimmune Diseases

Dysregulation of Tfh cell responses has been implicated in various autoimmune and inflammatory disorders in humans and mouse models. Thus, Tfh cell differentiation and maintenance must be closely regulated to ensure appropriate help to B cells (1, 4). Gensous et al. nicely recapitulated the molecular factors involved in Tfh cell formation in the context of a normal immune response, as well as markers associated with their identification (transcription factor, surface marker expression, and cytokine production). In their review article, Petersone et al., emphasized how CTLA-4-mediated regulation of CD28 signaling controls the engagement of secondary costimulatory pathways such as ICOS and OX40, and profoundly influences the T cell/B cell collaboration. Qin et al. summarized the recent findings of Tfh cell frequency and phenotype alteration in autoantibody-mediated diseases, such as systemic lupus erythematosus, Sjögren's syndrome, juvenile dermatomyositis, autoimmune myasthenia gravis, rheumatoid arthritis and type 1 diabetes.

Kim et al. recapitulated the molecular mechanisms for Tfh cell generation, survival and function in both humans and mice, and discussed the relationship between Tfh cells and autoimmune disease in animal models and patients. In their original research article, Felix et al. demonstrated that the P2RX7 receptor, an ATP-gated cation channel whose activation results in the release of pro-inflammatory molecules negatively controlling Tfh cell development in Peyer's patches, is required to control the autoimmune disease by keeping the Tfh cell response at check. Thus, contrary to exerting an anti-inflammatory effect, P2RX7 deficiency enhances autoimmune arthritis (Felix et al.). The results of the interesting clinical study by Cunill et al. demonstrated a pathogenic pro-inflammatory profile of circulating Tfh cells in relapsing–remitting multiple sclerosis patients, defined by high Tfh17/1 and low Tfh2 sub-populations, and the reversion of this biological signature by Dimethyl Fumarate treatment.

Serr and Daniel, in their brief review, reported the state of the art regarding the role of Tfh cells in the development of type 1 diabetes (T1D) and discussed thoroughly advances in the field of Tfh cell differentiation and function during the emergence of human islet autoimmunity. They also presented novel findings on the regulation of Tfh cells by microRNAs (miRNAs), as well as the potential use of miRNAs as biomarkers to predict disease progression. An analysis of Tfh cell frequencies or miRNAs involved in Tfh cell development in longitudinal samples could help in predicting the progression time to clinically overt T1D (Serr and Daniel). Interestingly, Cosorich et al.. provided evidence that Tfh cells expressing gut homing chemokine receptor CCR9 from the gastrointestinal tract exhibit plasticity and, by downmodulating the expression of CXCR5 can migrate to distal accessory organs of the digestive system, like the pancreas, where they may participate in autoimmunity.

An interesting review article about the emerging notion that an IL-1 axis, the key cytokine of innate immune responses predominantly produced by monocytes and macrophages, might control humoral immune responses by Tfh and Tfr cells was provided by Ritvo et al.. The discovery of a peculiar distribution of IL-1 receptors and IL-1 antagonists on Tfh and Tfr cells has led to a new understanding of the role of IL-1 in the control of antibody production. Some *in vitro* evidence confirmed a direct role of IL-1 in the activation of Tfh cells and indicated that targeting the IL-1 pathway should be important, although so far ignored, therapeutic approach to many autoimmune diseases, acting not just by reducing inflammation, but also by directly reducing the autoantibody response (Ritvo et al.).

## Tfh Cells in Chronic Inflammation

Lymphocytes migrating into chronically inflamed tissues form ectopic lymphoid structures with functional GCs, also known as tertiary lymphoid structures (TLS). T cells that interact with B cells in these sites, named Tfh-like cells, produce factors associated with B cell help, including IL-21 and the B cell chemoattractant CXCL13, yet vary dramatically in their resemblance to Tfh cells found in secondary lymphoid organs, e.g., surface phenotype, migratory capacity, and transcriptional regulation (10). The review article by Rao discusses observations from multiple diseases and models in which tissue-infiltrating T cells play a significant role in TLS formation. Hutloff also summarize findings on this topic discovered by studies on experimental animal models as well as some autoimmune and malignant diseases. Both reviews provide an interesting insight into a deeper understanding of these mechanisms in chronically inflamed tissues and suggest approaches to target these cells (Hutloff).

## Tfh Cells in Cancer

Interesting considerations also for cancer immunology have been generated from the comprehension of the mechanisms of Tfh cell development/maintenance. Accumulating evidence suggests that Tfh cells are involved in peripheral T cell and B cell-associated tumors, for example, in angioimmunoblastic T cell lymphoma (AITL), an aggressive tumor where neoplastic T cells express CXCL13, ICOS, CD154, CD40L, and NFATC1, making these T cells similar to Tfh cells. Follicular T cell lymphomas are another example, where infiltrating T cells resemble Tfh-like cells and express chemokines that play a role in the regulation of Treg and Th2 cell migration and modulate the activity of GC B cells (10–12). Moreover, the number of Tfr cells was found elevated during the various stages of the lymphoma development (Qin et al.).

On the other side, Tfh cells seem to have protective roles in some non-lymphoid tumors. Higher levels of Tfh cell infiltrates and an elevated presence of TLS within tumors have been associated with increased survival and reduced immunosuppression in patients with breast cancer. Evidence suggests that IL-21 and CXCL13 produced by tumor-infiltrating CD4 T cells may play a critical protective role. Infiltrating Tfh cells have also been reported in chronic lymphocytic leukemia, non-small cell lung cancer, osteosarcoma, and colorectal cancer, where, in some cases, they positively correlated with patient survival (Qin et al.). In their review article Poultsidi et al. raise the question of whether cancer neoantigens can drive Tfh differentiation. Another key question regards Tfh cell homing to lymph nodes and their role in tumor metastasis. Future research will help identify new molecular targets aiming at boosting Tfh cell responses against some types of tumors (Poultsidi et al.).

## Tfh Cells in Infections and Vaccine Responses

CD4^+^ T cell differentiation is influenced by a plethora of intrinsic and extrinsic factors and different classes of pathogens may induce a distinct balance of CD4^+^ T cell differentiation programs (9). Huang et al. recapitulated the molecular basis of virus-specific Tfh cells as part of a process involving multiple factors and stages and exhibiting distinct features. The original research article by Wang et al. demonstrated that the transcription factor T-bet, specifically expressed in type I Tfh cells, was dispensable for the early fate Tfh commitment, but essential for Tfh cell maintenance, proliferation and apoptosis inhibition during acute viral infection. The original research article by Danelli et al. reports an uncommonly strong bias toward Tfh cell differentiation of CD4^+^ T cells reactive with a retroviral envelope glycoprotein model antigen during retroviral infection. The response to the same antigen in different immunization regimens elicited a response typically balanced between Tfh and Th1 cells. Influencing factors for Tfh differentiation were T cell receptor (TCR) signaling that controlled PD-1 expression (Danelli et al.).

Several studies have revealed the important role of Tfh cells in Human Immunodeficiency Virus (HIV) pathogenesis. In the exciting research conducted by McCarty et al. on a Kenyan cohort of 76 perinatally HIV-infected children, HIV treatment-naïve children had reduced levels of cTfh cells compared to healthy children. Memory cTfh cells with elevated PD-1 levels correlated with advancing HIV disease status. Antiretroviral treatment restored cTfh cell frequency but did not decrease PD-1 levels on cTfh cells (Wang et al.). Greczmiel and Oxenius focused their review article on the mechanisms by which Tfh cells induce neutralizing protective antibody responses toward non- or poorly cytopathic viruses (i.e., HIV-1, HBV, HCV in humans, and LCMV in mice). These humoral responses are fundamental to afford control of the persistent infection—despite the risk of viral escape due to the high mutation rate during virus replication—in the absence of overt immunopathology (Greczmiel and Oxenius).

## Tfh Cells in Primary Immunodeficiencies (PIDs)

Several immunodeficiencies directly affect the development and functions of Tfh cells by impairing GC formation and altering B cell-dependent responses, e.g., mutations in SH2D1A, CD40L, ICOS, and STAT3 (6–8). In their perspective article, Preite et al. describe how PI3K-mediated pathways are likely to integrate multiple signals to promote Tfh cell differentiation, whose dysregulation is mirrored in human PID “Activated PI3K-delta Syndrome” (APDS). An original research article by Klocperk et al. described the number and phenotype of Tfh cells in a cohort of 17 patients with DiGeorge Syndrome, an immunodeficiency characterized by thymic dysplasia with increased susceptibility to infections and autoimmunity. While the population of cTfh cells was significantly expanded in patients with DiGeorge syndrome compared with age-matched healthy controls, their frequency did not significantly differ between DiGeorge patients with or without autoimmune manifestations, allergy, or dysgammaglobulinaemia. The authors concluded that the relative expansion of cTfh cells may be the result of impaired T cell development in patients with thymic dysplasia (Klocperk et al.).

## Tfh Cells in Transplantation Tolerance

The role of Tfh cells in transplantation is also a matter of great interest (3). In their original research article, Kwun et al. elucidated the post-transplant B cell immune response after T cell depletion. In a CD52 transgenic mouse model of heterotopic heart transplantation, the use of alemtuzumab, a monoclonal depleting antibody that binds to CD52 expressed on mature lymphocytes, promoted the production of serum donor-specific antibodies, allo-B cells and coronary allograft vasculopathy, a hallmark of chronic rejection. Moreover, hyperplastic GCs with elevated serum IL-21 were detected. The authors observed that the concomitant use of Anti-LFA-1 monoclonal antibody suppressed the humoral response in animals treated with alemtuzumab, providing a novel mechanism and paving the way to possibly new IL-21-directed therapeutic approaches for chronic antibody-mediated rejection (Kwun et al.).

## Follicular Regulatory T Cells (Tfr) in Health and Disease

Tfr cells are a recently identified subset of CD4^+^ FOXP3^+^ T cells that controls humoral immune responses in ectopic follicles and GCs of secondary lymphoid organs. Recent works have identified the functional and developmental characteristics of Tfr cells and have highlighted their characteristics of differentiation, GC recruitment and retention, and regulatory abilities. Moreover, Tfr cells finely regulate the balance of pathogen-specific to autoantibody production by constantly interacting with Tfh and B cell populations and altering their environment through cytokine production and sequestration, thereby influencing the quantity and quality of the GC response (1, 2).

In their review article, Fazilleau et al. focused on the role of Tfr cells as “negative regulators” dedicated to control the magnitude of the immune response in the GC, and thoroughly described the Tfr cell proprieties in the context of vaccination. On the same line, in their review, Miles and Connick summarize the current knowledge about Tfr cells in response to infection and their potential role in vaccine development. In the review article by Wing et al. the role of Tfr cells and the contribution activated extra-follicular Tregs (eTreg) in the control of humoral immunity, as well as the role of Tfr cells in autoimmune diseases and tumors, is summarized.

In the review article by Stebegg et al., an insightful overview of the complex and multilevel regulation of the GC is provided, including the biology of stromal cell subsets and chemokines network in both secondary lymphoid tissues and Peyer's patches. Xie et al. review article is focused on Tfr cell functions and discuss the evidence that Tfr cells can also play a major “helper” role in the GC-dependent antibody response by producing IL-10 that promotes GC B cell growth and high-affinity antibody production. Thus, in the context of the GC response, Tfr cells appear to maintain a key balance between help (GC maintenance, antibody response, and affinity) and suppression by controlling Tfh cell numbers, GC B cell numbers, Tfh cell cytokines, and autoantibodies (Xie et al.).

## Conclusions

Despite all the progress made in the last three decades, we are still at an early stage in our understanding of the sophisticated and multi-level role of Tfh and Tfr cells in health and disease. The complex niche of the GC is governed by delicate cognate interactions between Tfh, Tfr, B cells and stromal cells, the role and potential of the latter still need to be fully clarified. Evidence indicates that most patients affected by autoimmune diseases have increased numbers of Tfh cells that are also hyperactive, and possess altered numbers of Tfr cells with reduced function. Great interest is emerging on the role of Tfh and Tfr cells in PID and transplantation, where further studies may lead to the discovery of new therapeutic strategies and biological paradigms. Novel insights are also emerging on the role of Tfh and Tfr cells in tumors, allergy, infections, and vaccine responses that, together with the comprehension of the molecular mechanisms underlying the development and function of Tfh and Tfr cells in these clinical settings, may lead to the discovery of novel therapeutic targets. Increased knowledge of Tfh cells and Tfr cells has inspired, and hopefully it will continue to inspire more studies to reinstate the balance of these cells for the prevention and treatment of diverse human diseases.

## Author Contributions

MC, SS-A, and GF have made a substantial, direct and intellectual contribution to the writing of this editorial, and approved it for publication.

## Conflict of Interest

The authors declare that the research was conducted in the absence of any commercial or financial relationships that could be construed as a potential conflict of interest.
